# Acute Aortic Dissection Associated With Cancer Therapy in a Previously Normal Aorta

**DOI:** 10.1016/j.jaccas.2026.108471

**Published:** 2026-05-22

**Authors:** Simon A. Savage, Melissa Moore, Trevor Tnay, Elizabeth D. Paratz

**Affiliations:** a Department of Medicine, Bayside Health (Peninsula University Hospital), Frankston, Victoria, Australia; b Department of Cardiology, St Vincent's Hospital Melbourne, Fitzroy, Victoria, Australia; c Department of Oncology, St Vincent's Hospital Melbourne, Fitzroy, Victoria, Australia; d Department of Cardiothoracic Surgery, St Vincent's Hospital Melbourne, Fitzroy, Victoria, Australia

**Keywords:** aorta, cancer, dissection, imaging

## Abstract

**Background:**

Prophylactic management for aortic dissection, a life-threatening emergency, is traditionally guided by aortic diameter.

**Case Summary:**

A 60-year-old man, on pembrolizumab and lenvatinib for recurrent clear cell renal cell carcinoma, presented with chest pain, hypotension, and bradycardia during pembrolizumab infusion. Despite previously normal aortic dimensions on sequential imaging, computed tomography aortography revealed Stanford type A dissection requiring urgent surgery.

**Discussion:**

This case supports emerging evidence for an association between acute aortic syndromes and modern cancer therapies, even in patients without structural warning signs. The potential for acquired aortic wall disease without associated dilatation cautions against risk stratification based only on aortic dimensions.

**Take-Home Messages:**

Cancer therapy–related vascular toxicity may predispose to acute aortic syndromes through acquired aortic wall vulnerability, even in the absence of prior aortic dilatation. This highlights the limitations of diameter-based risk stratification and the need for vigilant clinical assessment and strict blood pressure control.

## Graphical Abstract

**Figure:**
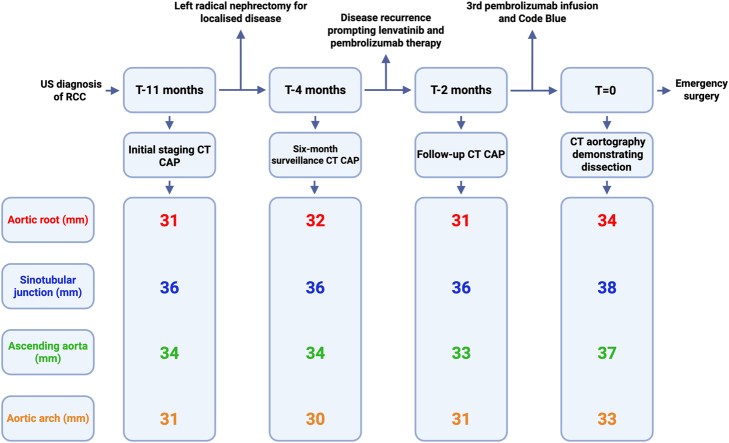

## History of Presentation

A 60-year-old Southeast Asian man presented with severe, sudden-onset, central chest pain radiating to the back during his third pembrolizumab infusion (programmed cell death protein 1 [PD-1] inhibitor) for recurrent clear cell renal cell carcinoma (ccRCC). A Code Blue emergency call (T = 0) was made due to hemodynamic instability, with systolic blood pressure of 60 mm Hg and heart rate of 35/min in sinus bradycardia.

## Past Medical History

Past medical history was significant for gastroesophageal reflux disease, active smoking, and acquired brain injury secondary to a workplace accident (still independent at home and retaining decision-making capacity). He was incidentally diagnosed with an 8-cm left renal cell carcinoma during ultrasound investigation of epigastric discomfort. Initial computed tomography (CT) scan (T-11 months) demonstrated no nodal or metastatic disease, and he underwent laparoscopic left radical nephrectomy. Pathological analysis confirmed a high-grade ccRCC confined to the kidney with clear resection margins. Six-month surveillance CT (T-4 months) indicated extensive retroperitoneal and intraabdominal nodal dissemination prompting treatment with pembrolizumab and lenvatinib (multikinase inhibitor) with anticipated prognostic benefit. Follow-up imaging (T-2 months) demonstrated marginal intraabdominal progression and new 3-mm right upper lobe nodules, suggestive of pulmonary metastasis. Serial CT imaging over 9 months (T-11 months to T-2 months) demonstrated stable and normal ascending aortic dimensions, without evidence of progressive enlargement, aneurysm development, or imaging features suggestive of aortitis.

**Visual Summary:**
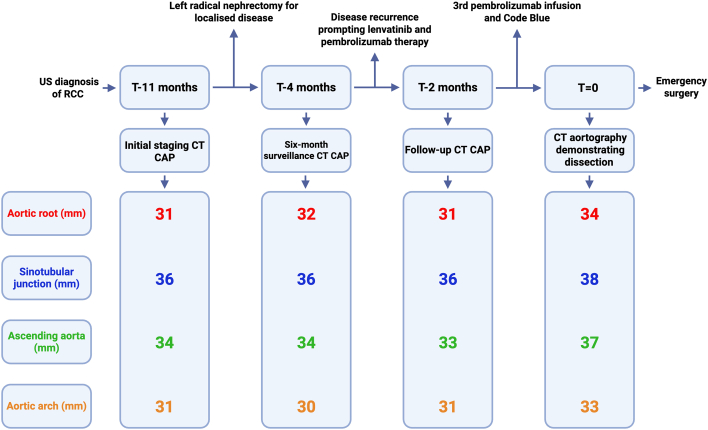
Event Timeline With Sequential Aortic Dimensions This figure depicts important clinical events preceding the patient's presentation with chest pain and Code Blue. It also demonstrates stable and normal oblique aortic diameters with subsequent dilatation at all levels during acute dissection. CT CAP = computed tomography of chest/abdomen/pelvis; RCC = renal cell carcinoma; US = ultrasound.

## Investigations

CT aortography performed to investigate his severe chest pain (T = 0) demonstrated Stanford type A aortic dissection of the entire aorta with maximal axial diameter of 50 mm in the ascending aorta, and acute enlargement in all oblique measurements: aortic root 34 mm, sinotubular junction 38 mm, ascending aorta 37 mm, and aortic arch 33 mm (Figure 1). The dissection flap extended into the right common and internal carotid arteries causing new occlusion without symptoms of malperfusion.

**Figure 1:**
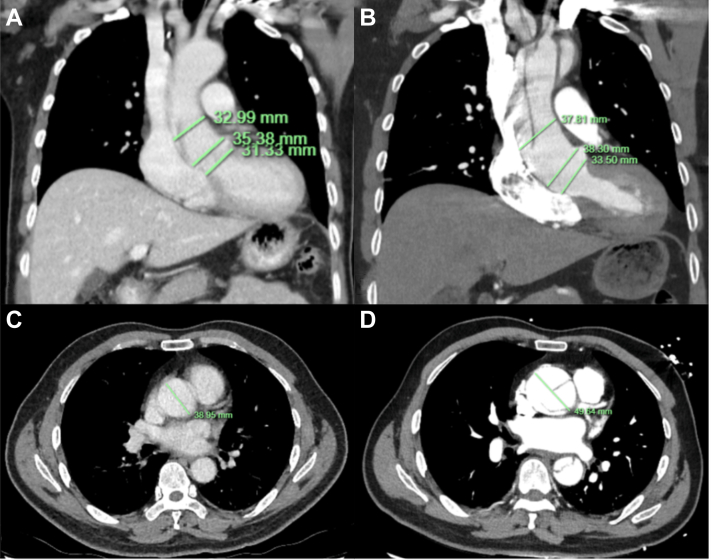
Sequential Computed Tomography Images With Aortic Measurements Coronal computed tomography images before (A) and during (B) dissection demonstrate acute dilatation in all oblique aortic measurements. Axial ascending aortic measurements before (C) and during (D) dissection demonstrate acute dilatation of 11 mm to a diameter of 50 mm.

## Management

The patient underwent urgent replacement of the ascending aorta and hemiarch with aortic valve repair. The aorta was described as fragile, but no tissue specimens were submitted for histopathological analysis. There was no radiologic or surgical evidence of pericardial effusion and no concern for cardiac tamponade. Carotid extension of the dissection was not managed surgically.

## Outcome and Follow-Up

He was admitted to the intensive care unit postoperatively where he suffered delirium, acute kidney injury, liver function derangement, and sepsis. He developed left hemineglect on postoperative day 2 and was diagnosed with an ischemic right middle cerebral artery stroke attributed to an embolic event from dissection given recanalization of previously occluded carotid arteries on repeat imaging. The stroke team determined that he was not a candidate for reperfusion therapy and he was treated with aspirin and prophylactic enoxaparin. He was extubated on postoperative day 3 and transferred to the ward the following day. His progress was further complicated by residual Stanford type B dissection, managed conservatively, as well as hemorrhagic transformation of the cerebral infarct, prompting temporary discontinuation of aspirin and prophylactic enoxaparin. He was eventually transferred to rehabilitation on postoperative day 22 with multidisciplinary follow-up in cardiology, cardiothoracic surgery, vascular surgery, and stroke outpatient clinics. Pembrolizumab and lenvatinib were withheld at discharge pending a further CT chest/abdomen/pelvis planned for 6 weeks postdissection.

## Discussion

This case highlights the potential association between modern oncological therapies and acute aortic syndromes, as well as the limitations of diameter-based risk stratification.

Aortic dissection is a life-threatening emergency associated with high morbidity and mortality. Major risk factors include aortic dilatation, bicuspid aortic valve, hypertension, smoking, aortitis, and genetic aortopathies.^1^ Aortic size has traditionally been used as the primary predictor of dissection and rupture, with prophylactic intervention thresholds determined largely by aortic diameter.^1^

Pembrolizumab is a novel monoclonal antibody that blocks the PD-1 receptor on T cells, removing inhibitory immune checkpoints and restoring antitumor activity.^2^ It has been used for contemporary management of many cancers.^2^ Although pembrolizumab causes fewer side effects than chemotherapy, it is associated with a number of immune-related adverse events due to its role as a checkpoint inhibitor including pneumonitis, hypophysitis, thyroiditis, colitis, hepatitis, nephritis, and type 1 diabetes mellitus.^2^ Of note, it also has been associated with aortitis, which can lead to acute aortic syndromes through several mechanisms including degeneration of the medial layer with elastin disruption and wall dilatation.^3^

Lenvatinib is a multiple receptor tyrosine kinase inhibitor that inhibits angiogenesis required for tumor oxygenation, nutrition, and growth.^4^ It also has been associated with aortic dissection through different mechanisms to pembrolizumab, including hypertension and vascular endothelial growth factor inhibition-related interference with aortic wall structure.^4^ Therapy-induced hypertension may represent a key mechanistic contributor, increasing aortic wall stress and potentially accelerating medial layer injury in susceptible patients. Interestingly, a recent preclinical study of murine and porcine abdominal aortic aneurysm models as well as human-derived cell data suggested that lenvatinib may halt aortic aneurysm growth by improving vascular smooth muscle contractility.^5^ Given that aneurysm expansion is a major risk factor for dissection, this suggests a more nuanced relationship between the drug and dissection.

Understandably, neither PD-1 inhibitor– nor multikinase inhibitor–associated aortic disease have been studied with major clinical trials, but associations have been described in several case reports with suggestion of a potential causal link.^3^^,^^4^^,^^6^^,^^7^ Sultan et al^3^ described intramural hematoma with rupture of the thoracic aorta during combination therapy with pembrolizumab and axitinib, a multikinase inhibitor similar to lenvatinib.^4^ Despite the absence of histopathological evidence to support aortitis in the current case, a plausible association is suggested due to biological feasibility, the absence of other significant risk factors besides smoking, the documented preceding normal aortic dimensions, and the temporal association of pembrolizumab infusion and acute dissection in the context of ongoing lenvatinib therapy. Possible aortic complications of PD-1 inhibitors and multikinase inhibitors are described in Figure 2.

**Figure 2:**
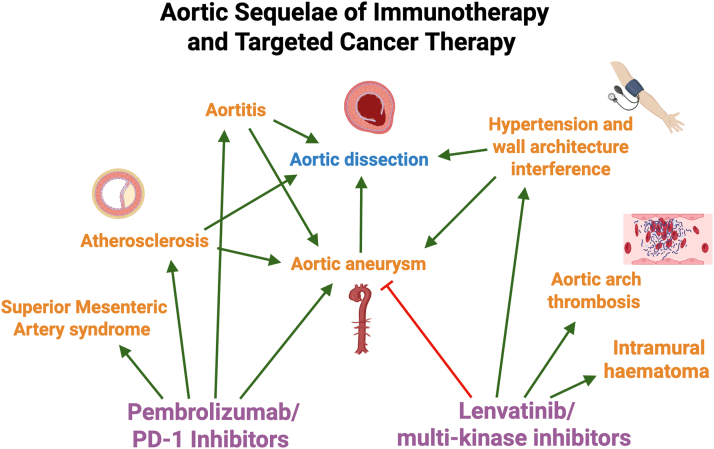
Possible Aortic Sequelae of Immunotherapy and Targeted Cancer Therapy This figure explores possible aortic sequelae following the use of PD-1 (programmed cell death 1) inhibitors and multikinase inhibitors, drawing on evidence from published case reports and known pathophysiological pathways. Green arrows denote causative pathways and red arrows denote inhibitory pathways.

This case is distinctive in offering sequential aortic imaging as part of the diagnosis and monitoring of ccRCC. The stable aortic size before dissection aligns with national pre-dissection echocardiographic data from Paratz et al,^8^ which suggested that up to 97% of aortic dissections occurred in nonseverely dilated aortas.^8^ These findings reinforce that intrinsic or acquired aortic wall disease, rather than diameter alone, may be a key determinant of acute aortic syndromes. Oncological therapies may predispose to acquired wall vulnerability through endothelial dysfunction, medial layer injury, and hypertension, rather than progressive dilatation detectable through traditional monitoring. This merits further investigation of risk prediction methods for acute aortic syndromes in patients receiving immune checkpoint and multikinase inhibitors including genetic and family profiling, machine learning, biomarker analysis of vascular injury, and radiographic assessment of wall integrity and flow dynamics. Clinicians should consider strict blood pressure control and maintain clinical vigilance for acute aortic syndromes in patients receiving these therapies.

## Conclusions

This case suggests a possible association between cancer therapy and acute aortic syndromes, emphasizing the role of acquired aortic wall vulnerability and the limitations of diameter-based risk stratification.

## Funding Support and Author Disclosures

The authors have reported that they have no relationships relevant to the contents of this paper to disclose.

**Take-Home Messages**

• Cancer therapy–related vascular toxicity may predispose to acute aortic syndromes through acquired aortic wall vulnerability, even in the absence of prior aortic dilatation.
• This highlights the limitations of diameter-based risk stratification and the need for vigilant clinical assessment and strict blood pressure control.
